# Multiple Lineages of Usutu Virus (*Flaviviridae*, *Flavivirus*) in Blackbirds (*Turdus merula*) and Mosquitoes (*Culex pipiens*, *Cx. modestus*) in the Czech Republic (2016–2019)

**DOI:** 10.3390/microorganisms7110568

**Published:** 2019-11-16

**Authors:** Vaclav Hönig, Martin Palus, Tomas Kaspar, Marta Zemanova, Karolina Majerova, Lada Hofmannova, Petr Papezik, Silvie Sikutova, Frantisek Rettich, Zdenek Hubalek, Ivo Rudolf, Jan Votypka, David Modry, Daniel Ruzek

**Affiliations:** 1 Institute of Parasitology, Biology Centre, Czech Academy of Sciences, 37005 Ceske Budejovice, Czech Republic; palus@paru.cas.cz (M.P.); tomas18@seznam.cz (T.K.); marta.zemanova@paru.cas.cz (M.Z.); karolina.majerova@natur.cuni.cz (K.M.); jan.votypka@natur.cuni.cz (J.V.); modryd@vfu.cz (D.M.); ruzekd@paru.cas.cz (D.R.); 2 Department of Virology, Veterinary Research Institute, 62100 Brno, Czech Republic; 3 Faculty of Science, University of South Bohemia, 37005 Ceske Budejovice, Czech Republic; 4 Department of Parasitology, Faculty of Science, Charles University, 12800 Prague, Czech Republic; 5 Department of Pathology and Parasitology, Faculty of Veterinary Medicine, University of Veterinary and Pharmaceutical Sciences, 61242 Brno, Czech Republic; Lada.Hurkova@seznam.cz (L.H.); Reptimania@email.cz (P.P.); 6 Institute of Vertebrate Biology, Czech Academy of Sciences, 60365 Brno, Czech Republic; sikutova@ivb.cz (S.S.); zhubalek@brno.cas.cz (Z.H.); rudolf@ivb.cz (I.R.); 7 Centre for Epidemiology and Microbiology, National Institute of Public Health, 10000 Prague, Czech Republic; rettich@szu.cz; 8 CEITEC, University of Veterinary and Pharmaceutical Sciences, 61242 Brno, Czech Republic

**Keywords:** Usutu virus, blackbird, mosquito, *Culex* spp.

## Abstract

Usutu virus (USUV) is a flavivirus (*Flaviviridae*: *Flavivirus*) of an African origin transmitted among its natural hosts (diverse species of birds) by mosquitoes. The virus was introduced multiple times to Europe where it caused mortality of blackbirds (*Turdus merula*) and certain other susceptible species of birds. In this study, we report detection of USUV RNA in blackbirds, *Culex pipiens* and *Cx. modestus* mosquitoes in the Czech Republic, and isolation of 10 new Czech USUV strains from carcasses of blackbirds in cell culture. Multiple lineages (Europe 1, 2 and Africa 3) of USUV were found in blackbirds and mosquitoes in the southeastern part of the country. A single USUV lineage (Europe 3) was found in Prague and was likely associated with increased mortalities in the local blackbird population seen in this area in 2018. USUV genomic RNA (lineage Europe 2) was detected in a pool of *Cx. pipiens* mosquitoes from South Bohemia (southern part of the country), where no major mortality of birds has been reported so far, and no flavivirus RNA has been found in randomly sampled cadavers of blackbirds. The obtained data contributes to our knowledge about USUV genetic variability, distribution and spread in Central Europe.

## 1. Introduction

Usutu virus (USUV) is a flavivirus (family *Flaviviridae*, genus *Flavivirus*) transmitted by ornithophilic mosquitoes. The virus was isolated from a number of mosquito species of the genera: *Aedes*, *Anopheles*, *Culex*, *Culiseta*, *Ochlerotatus* summarized in [1]), hence they are considered as potential vectors. Nevertheless, vector competence was experimentally proven only for *Culex* spp. [2,3,4,5]. A variety of resident (e.g., Eurasian magpie (*Pica pica*), domestic pigeon (*Columba livia domestica*), hooded crow (*Corvus cornix*), carrion crow (*Corvus corone*), Eurasian nuthatch (*Sita europea*), great tit (*Parus major*)) and partially migratory or migratory (e.g., Euroasian blackbird (*Turdus merula*), song thrush (*T. philomelos*), white stork (*Ciconia ciconia*), common redstart (*Phoenicurus phoenicurus*), and European robin (*Erithacus rubecula*)) bird species are considered the probable natural (reservoir) hosts in Europe, based on serologic evidence and direct proof of the virus [1,6,7], although the virus was also found in other vertebrates, such as bats [8], rodents and a shrew [9]. Passeriform and strigiform birds are particularly sensitive to the infection in the sense of symptomatic disease (symptoms include: featherless heads, apathy, inability to fly, incoordination, splenomegaly, necrosis in liver and spleen, and signs of encephalitis) frequently followed by death [10,11,12,13,14]. Infections of humans are mostly associated with asymptomatic course [15,16,17] or mild symptoms including fever, rash or jaundice [18]. Predominantly although not exclusively, in immunocompromised patients, USUV may cause infections of central nervous system [19,20,21].

Like other flaviviruses, the genomic +ssRNA of USUV encodes three structural proteins forming viral capsid (protein C), membrane (prM/M) and envelope (E). The remaining part of the genome encodes seven non-structural proteins (NS1, NS2A, NS2B, NS3, NS4A, NS4B, and NS5) [22,23]. Protein E as a major surface-exposed protein plays a crucial role in the virus-host cell and virus-immune system interactions. NS5 protein is a viral RNA-dependent RNA polymerase and methyltransferase [24].

Usutu virus was first isolated in African Swaziland from a mosquito *Cx. naevei* in 1959 [25]. In Europe, the so far oldest documented occurrence of USUV is dated back in 1996. It was retrospectively detected in a dead blackbird from Italy [26]. The first large outbreak among blackbirds occurred in Austria in 2001 [27]. Thereafter, the virus was repeatedly introduced to Europe from Africa most probably by migratory birds and established diverse genetic lineages in Europe [23]. Currently, the occurrence of multiple lineages of USUV is reported in various biological samples (birds, mosquitoes, human) from 15 European countries [28]. In the Czech Republic, two strains of USUV (both lineage Europe 1) were isolated from dead blackbirds found in 2011 and 2012 in the city of Brno (South Moravia—south-eastern part of the country neighboring with Austria) [29] and in a pool of *Cx. modestus* mosquitoes sampled in 2013 in South Moravia [30].

There are reports of multiple USUV outbreaks mainly among blackbirds from other European countries e.g., [10,11,31,32,33]; however, information about USUV spread and genetic heterogeneity in the Czech Republic is limited. Since there are substantial changes in the distribution of different genetic lineages of USUV currently occurring throughout Europe, the information about the circulating strains and lineages in the individual European countries is of high importance. In this study, we tested the samples of dead blackbirds found during a season of increased blackbird mortality in the outskirts of Prague city in 2018 and randomly sampled in two other cities (Brno and Ceske Budejovice) in the Czech Republic in 2017–2019 for the presence of USUV RNA. Herein, we report a detection of multiple USUV lineages in blackbirds, *Culex pipiens* and *Cx. modestus* mosquitoes in the Czech Republic, and isolation of 10 USUV strains from carcasses of blackbirds using cell culture. This data contributes to our knowledge about USUV genetic variability, distribution and spread in Central Europe.

## 2. Materials and Methods

### 2.1. Sample Collection

**Figure 1:**
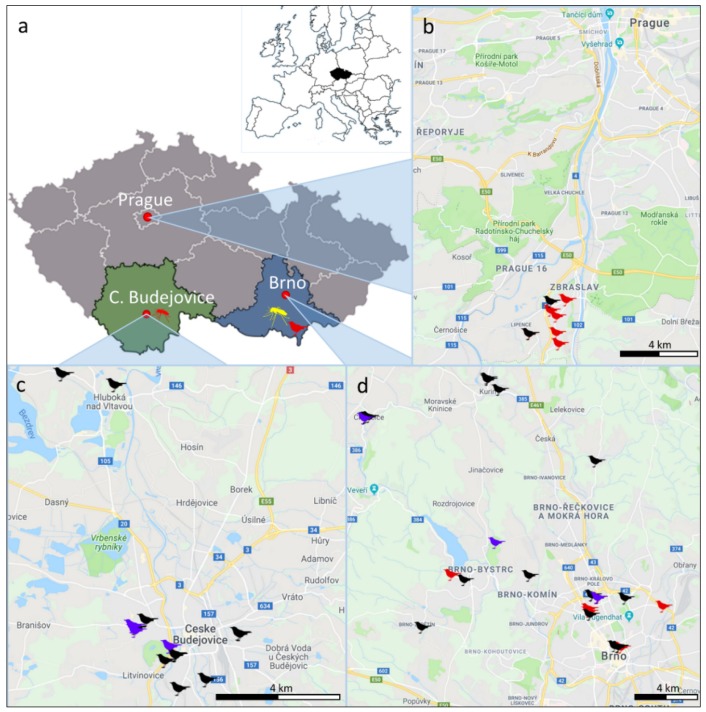
Localization of the cadavers of blackbirds (*Turdus merula*) and song thrushes (*T. philomelos*) collected in 2017–2019 in the three geographical locations in the Czech Republic (indicated in black in the overview map of Europe) and used for detection of Usutu virus (USUV). (**a**) Localization of the three main sampling sites (cities of Prague, Brno and Ceske Budejovice) and localization of the three additional USUV positive samples collected outside the area of the three cities are depicted in the map of the Czech Republic. The region of South Moravia is labeled by a blue background and the region of South Bohemia by green. Bird pictogram indicates an USUV positive blackbird, collected in the town of Breclav-South Moravia); mosquito pictograms indicate USUV positive mosquito pools (pool of *Cx. modestus* collected near Hlohovec-South Moravia in yellow, *Culex pipiens* collected near Lomnice nad Luznici-South Bohemia in red). Position of cadavers of blackbirds and song thrushes collected in the three cities are presented in the remaining maps: Prague (**b**), Ceske Budejovice (**c**), Brno (**d**). Blackbirds negative for USUV genomic RNA are indicated in black, USUV positive blackbirds in red, and song thrushes (all USUV negative) in violet.

Cadavers of blackbirds (*Turdus merula*) were collected in three cities (and their surroundings) in the Czech Republic: Prague (2018), Brno (2017–2019) and Ceske Budejovice (2017) (Figure 1). In Prague, the blackbird cadavers were collected during a local outbreak of increased mortality, when tens of dead blackbirds were found in a period of several weeks in a specific area of approximately 2 km^2^. Cadavers from Brno and Ceske Budejovice were acquired within a project focused on the use of accidentally killed urban free-living animals for monitoring of tick-borne diseases and thus sampled randomly. Apart from blackbirds, 10 song thrushes (*T. philomelos*) were collected and analyzed (Table 1).

The cadavers had been exposed for an unknown time to outdoor temperatures before collection. The place, time, date of collection, and conjectured cause of death were recorded. Cadavers were stored at −80 °C until necropsied.

### 2.2. Sample Processing and RNA Extraction

Samples of brain, liver, muscle, and blood were collected using sterile instruments for each tissue in the individual cadavers. Two specimens of each tissue (for RNA extraction and virus isolation) were created and stored at −80 °C or processed immediately. One specimen of each tissue (brain, liver, muscle) was used to prepare 30% (*w*/*v*) suspension in lysis RLT buffer (Qiagen, Hilden, Germany) containing beta-mercaptoethanol. Stainless-steel beads of 5-mm (Qiagen) and Tissue Lyzer II (Qiagen) were used for tissue homogenization. After mechanical disruption (30 Hz for 30 s for liver and 2 min for muscle and brain tissue), the homogenate was digested with 20 µL of proteinase K for 30 min at 56 °C. The lysate was clarified by centrifugation and supernatant was collected. The blood samples were resuspended directly in 560 µL of AVL buffer. From each sample, 140 µL were used for RNA isolation using the Qiamp Viral RNA Mini Kit (Qiagen) according to manufacturer’s instructions, modified as follows. On column RNase-free DNase I (Qiagen), treatment was performed for 15 min at room temperature after the first washing step and an additional AW1 wash step was included after the digestion. The RNA was eluted with 60 µL of AVE buffer. Subsequently, the membrane was washed once more with the eluate to increase the yield of RNA. Spare aliquots of PCR positive tissue samples were used for virus isolation attempts in cell cultures.

### 2.3. Screening for flavivirus RNA

The isolated RNA was used as a template for one-step RT-PCR (OneStep RT-PCR Kit, Qiagen). The reaction was performed according to manufacturer’s instructions in a total volume of 25 µL. Universal flavivirus primers [34] in 0.4 µM final concentration and 5 µL of isolated RNA were used. The reverse transcription and PCR were performed in a thermal cycler using the following program: 50 °C, 60 min-reverse transcription; 95 °C, 15 min—enzyme activation, followed by 40 cycles of 95 °C, 30 s—denaturation; 57 °C, 30 s—primer annealing; 72 °C, 1 min—extension, the program was completed by final extension 72 °C, 3 min. The RT-PCR products (10 µL of each reaction) were analyzed by agarose gel electrophoresis. PCR products were enzymatically purified (Exonuclease I FastAP™ Thermosensitive Alkaline Phosphatase, Thermo Fisher Scientific, Waltham, MA, USA) and submitted for sequencing from both sides to confirm the identity of the virus.

### 2.4. Sequencing and Phylogenetic Analyses

Samples positive for USUV genomic RNA (RNA isolated from the original tissue homogenates) were subjected to cDNA synthesis using the 1st Strand cDNA Synthesis Kit (Roche, Basel, Switzerland) according to the manufacturer’s instructions. Random hexamer primers and 4 µL of template total RNA were used for reverse transcription. Selected portions of the USUV genome were sequenced using Sanger sequencing primer walking strategy (primer nucleotide sequences are presented in Table S1). Three additional samples were obtained during mosquito and bird surveillance for West Nile virus [35,36] and previously found to be USUV RNA positive. The samples were sequenced as described above and included in the phylogenetic analysis: sample 2Cx_136 comprised of a pool of 42 *Cx. pipiens* females sampled in South Bohemia, near Lomnice nad Luznici, in 2018; sample 3Cx_16-99 was obtained from a pool of 52 *Cx. modestus* females sampled in South Moravia, near Hlohovec, in 2016 and sample 1TM10Bre was obtained from a brain tissue of a dead blackbird found in South Moravia, town of Breclav in 2018 (Figure 1). The nucleotide sequences obtained in our study were submitted to GenBank under the following accession numbers: NS4B/NS5 section: MN384964, MN395369, MN419895-MN419913; prM/M/E section: MN395370-MN395384 (Table S2).

Geneious Prime, version 2019.0.4, including available plugins was used for manual control, analysis and assembly of the nucleotide sequences as well as for alignment of the sequences, calculations of genetic distance, and constructions of phylogenetic trees. The sequences were aligned using MAFFT, version 7, and FTT-NS-i x1000 algorithm [37,38] and identical sequences were removed. Suitable substitution models were identified based on Akaike and Bayesian information criterion using jModelTest 2.1.9 [39]. Phylogenies were inferred based on Maximum likelihood (PHYML 3.3.20180621) [40] and Bayesian Markov Chain Monte Carlo (Mr Bayes 3.2.6) [41] approach in parallel.

### 2.5. Virus Isolation in Cell Culture

Brain samples of the USUV RNA positive individuals were used for isolation of the replicating virus using cell cultures. Mammalian PS (porcine kidney) [42] (Kozuch and Mayer, 1975) and mosquito C6/36 cells [43,44](Igarashi, 1978; Singh, 1967) were seeded 1.5 million cells per well in a 6-well plate in 3 mL of PS medium (Leibowitz medium (L-15), 3% precolostral calf serum, 1% l-glutamine, 100 U/mL penicillin, and 100 μg/mL streptomycin) or C6/36 medium (L15, 10 % fetal bovine serum, 5 % tryptose phosphate broth, 1% l-glutamine, 100 U/mL penicillin, and 100 μg/mL streptomycin). The cells were cultivated for 1 day in 0.5% of CO_2_ at 37 °C (PS) or 28 °C (C6/36), respectively. Brain tissue samples were homogenized in the culture media using stainless-steel beads for 1 min 30 Hz in cooled blocks of Tissue Lyzer II (Qiagen) to prepare a 30% (*w*/*v*) suspension. The suspension was clarified by centrifugation (10 min 33,000× *g* at 4 °C), supernatant was filtered using 0.2 µm syringe filters (VWR), and 100 µL of the filtrate were added to the prepared cells in the plate. The same volume of media used for homogenization was added in negative control wells. After 3 h of incubation at appropriate temperature (0.5% of CO_2_), the medium was removed, cells were washed by 3 mL of phosphate-buffered saline (PBS), 3 mL of fresh medium was added, and the cells were further cultivated. On days 1, 2, and 5 post infection, 2 mL of culture medium were sampled and replaced by fresh medium. The identity of the virus was confirmed by RT-PCR and sequencing. Virus titers were determined by plaque assay using PS cell culture, and a virus antigen was detected using a flavivirus-specific immunofluorescence staining, as described previously [45].

## 3. Results

### 3.1. Screening for USUV RNA

From the total of 55 blackbirds (*T. merula*), 20 (36%) were positive for flavivirus RNA. All positive findings were subsequently confirmed as USUV by sequencing and Blast analysis. From the total of eight individuals collected in the area of Prague, six were positive for USUV RNA. Another 14 positive blackbirds (17%) were found among randomly sampled bird cadavers in the area of Brno, whereas no positive animal was acquired in Ceske Budejovice. Of the 14 positive individuals from Brno, 9 were sampled in 2017 and 5 in 2018. All of the USUV positive blackbirds had detectable viral RNA in brain tissue. Detailed results including a comparison of different tissues are summarized in Table 2 and Table S3. None of the 10 song thrushes (*T. philomelos*) were positive for flavivirus RNA.

### 3.2. Sequence Analysis

We retrieved continuous nucleotide sequences from 18 of 21 positive blackbirds and both of the infected mosquito pools (from the original tissue/mosquito pool homogenates). From the prM/E protein section, we have determined an almost complete coding sequence of prM, whole coding sequence of M protein and E protein (nucleotide positions 562–2475). Furthermore, partial nucleotide coding sequences for viral NS4B peptide and complete coding regions of protein NS5 were determined (nucleotide positions 7372–10,398). In the case of the sample 001TM10Bre acquired from a dead blackbird from South Moravia in 2018, only the complete NS4B/NS5 section was obtained.

**Figure 2:**
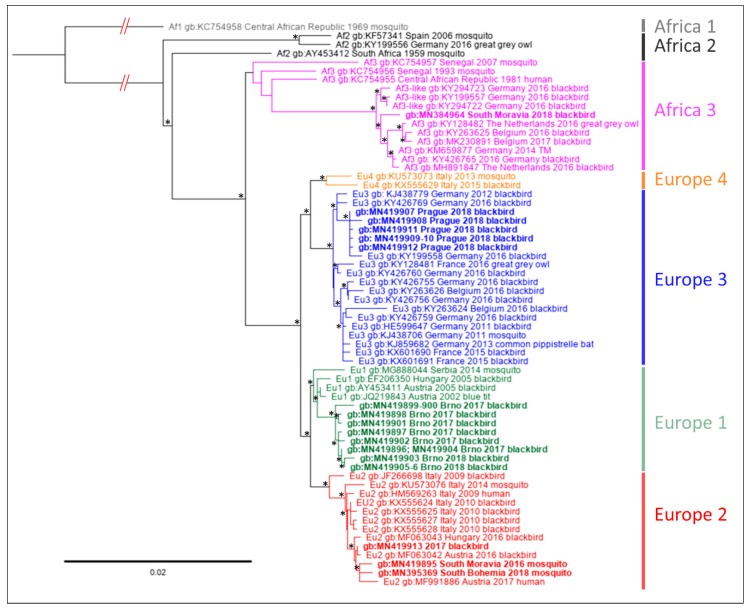
Phylogenetic maximum likelihood tree based on partial NS4B-coding nucleotide sequence and complete-coding sequences of NS5 protein of Usutu virus (nucleotide positions 7372–10,398 according to complete genome sequence of Vienna strain, AY453411). Sequence Africa 1, AY453412 was used as the outgroup. The code of the sequences consists of GenBank accession number, place and year of origin. The sequences obtained in this study are indicated by bold; individual geographical lineages are color-coded). The tree was generated using the GTR+I+G substitution model and 1000 replicates bootstrap analysis. Nodes with bootstrap support of <50% are indicated by asterisk. The lengths of tree branches correspond to the number of substitutions per site.

**Figure 3:**
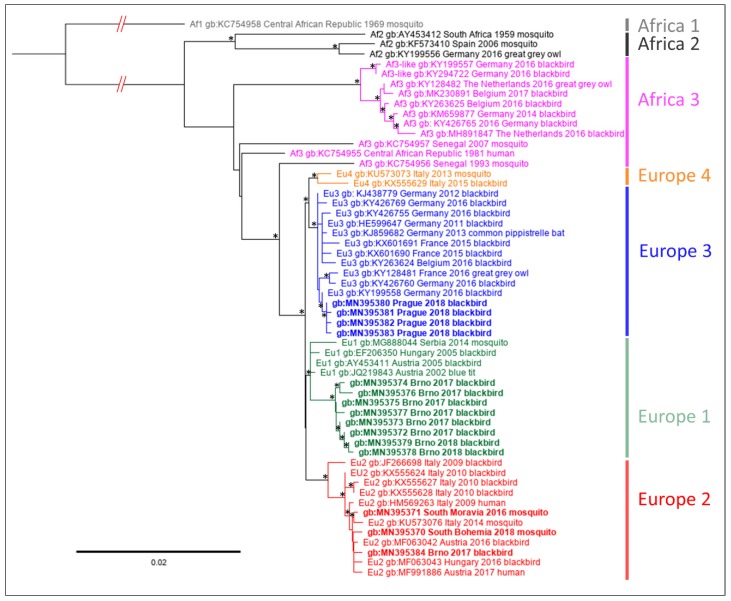
Phylogenetic maximum likelihood tree based on partial nucleotide sequence of prM protein and complete coding sequences of M and E proteins of Usutu virus (nucleotide positions 562–2475 according to complete genome sequence of Vienna strain, AY453411). Sequence Africa 1, AY453412, was used as the outgroup. The code of the sequences consists of GenBank accession number, place and year of origin. The sequences obtained in this study are indicated by bold; individual geographical lineages are color-coded. The tree was generated using the GTR+I substitution model and 1000 replicates bootstrap analysis. Nodes with bootstrap support of <50% are indicated by asterisk. The lengths of tree branches correspond to the number of substitutions per site.

The nucleotide sequences described above were used for phylogenetic analyses in order to assign them to established genetic lineages of the virus [31,46,47]. Using the two genomic loci, multiple datasets and phylogenetic approaches, the sequences obtained from samples from Prague (2018) clustered consistently into lineage Europe 3, whereas most sequences from blackbirds collected in Brno (2017, 2018) were associated with lineage Europe 1. Single USUV strain detected in a *T. merula* collected in Brno and sequences obtained from both the USUV positive mosquito pools were placed into lineage Europe 2 (Figure 2 and Figure 3; phylogenetic trees reconstructed based on Bayesian inference are available as supplementary data: Figures S1 and S2). There were only negligible differences in the detailed topology in the phylogenetic tree based on prM/E compared to NS4B/NS5 protein-coding sequences. The position of Africa 3 lineage strains acquired in Africa was to a certain level unstable. Nevertheless, the NS4B/NS5 sequence obtained from a blackbird collected in South Moravia clustered consistently with the Africa 3 sequences of the West European origin (Figure 2 and Figure S1).

### 3.3. Usutu Virus Isolation

**Figure 4:**
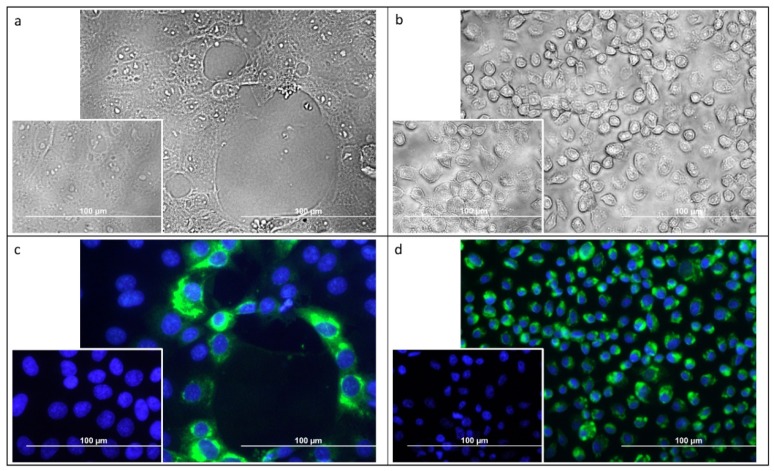
Mammalian, porcine kidney (PS) (**a**,**c**) and mosquito C6/36 cells (**b**,**d**) infected by the strains of Usutu virus isolated from dead blackbirds in this study. Infected cell cultures are presented in the large images and uninfected control cells in smaller images (3 days post-infection). Cytopathic effect was observed in infected PS cells (**a**) whereas no such changes were seen in C6/36 mosquito cells (**b**). Flaviviral E protein antigen was labeled using indirect immuno-fluorescence (green) in PS (**c**) and C6/36 cells (**d**). Cell nuclei were counterstained using DAPI (blue).

Selected homogenates of RT-PCR positive samples were used for isolation of the virus using mammalian (PS) and mosquito (C6/36) cell lines. We have isolated USUV in 10 out of 12 samples (six of Europe 1 lineage and four of Europe 3) with the same results obtained in both cell culture systems. The active replication of the virus was confirmed using plaque assay (all the successfully isolated viral strains were obtained in both-PS and C6/36 cell lines). In PS cells, average virus titer in culture media reached 4.83 ± 0.79 log10 pfu/mL on day-2 post-infection (p.i.) and 2.27 ± 1.11 log10 pfu/mL on day-5 p.i. In C6/36 cells, average virus titer in culture media reached 3.11 ± 1.37 log10 pfu/mL on day 2 p.i. and 6.96 ± 0.61 log10 pfu/mL on day 5 p.i. In PS cells, the viral strains generated a cytopathic effect, whereas in mosquito C6/36 cells, no cytopathic effect was observed (Figure 4a,b). Immunofluorescence staining of viral E antigen revealed that only a low percentage of PS cells in culture are infected, whereas almost all C6/36 cells in the culture are antigen-positive. For example, in the case of strain 202TM10, 13.3% of PS cells were infected on day 3 p.i. compared to 95.5% of C6/36 cells (average of 10 fields with >50 cells) (Figure 4c,d).

All the isolated strains were deposited in the Arbovirus collection of the Biology Centre Collection of Organisms (BCCO) under following identification numbers: BCCO_50_0480 to BCCO_50_0501 [48] (Table S2).

## 4. Discussion

In the Czech Republic, the occurrence of USUV has been reported in isolated cases and restricted to the southeastern part (South Moravia) of the country [29,30] neighboring with an USUV endemic region in Austria [14,31]. Our study was initiated by an observation of increased mortality among blackbirds outside this area in the city of Prague (central part of the country). USUV RNA was demonstrated in multiple tissues of dead blackbirds (in six out of eight individuals) sampled in the area. These USUV strains were most closely related to a strain of the Europe 3 lineage (KY199558) involved in an outbreak in eastern Germany in 2016 [33].

Interestingly, we were able to detect USUV RNA also in randomly collected dead blackbirds in the city of Brno in the area of previous occurrence, where, however, no mass mortality of blackbirds was observed. Phylogenetic analyses showed that these viral strains belong to lineages Europe 1 and 2. The Europe 1 lineage virus was found previously in this region [30] and both lineages co-circulate in neighboring Austria [31,49]. In Austria, it was described previously that USUV outbreaks among blackbirds resulted in a certain level of herd immunity, when increased seroprevalence in bird population resulted in decreased numbers of acutely infected individuals, or decreased viral loads in acutely infected birds in the years following an outbreak [50]. Nevertheless, this scenario did not take place in the more recent outbreaks in Germany [11,32]. As USUV was found repeatedly since 2010 in South Moravia [29,30], it is likely endemic to the region and possibly controlled by herd immunity in the terms of decreasing the frequency of infected birds as the source of the virus for the vectors and susceptible individuals as new potential USUV hosts. Whereas, the Europe 3 lineage USUV was probably newly introduced to the area of Prague in 2018 and caused a local outbreak, which is similar to the situation observed in eastern Germany in the same year [32].

In some areas, USUV probably may circulate unnoticed without causing any major mortalities in (urban) populations of birds. This hypothesis is supported by the detection of the virus in a pool of mosquitoes collected in South Bohemia, the southwestern part of the country close to the city of Ceske Budejovice, where no USUV positive birds were found and no mass mortality among birds was noticed.

For the first time, an USUV strain of Africa 3 lineage was found in the Czech Republic in a dead blackbird from South Moravia region (found in the town of Breclav). USUV strains of Africa 3 and Africa 3-like lineages were repeatedly detected in Europe [46] including recent reports from eastern Germany [32,33]. Nevertheless, Austria is a probable source of the virus recorded in our study as this USUV lineage is also present in the area neighboring with South Moravia [15,49]. Unfortunately, no suitable sequences were available from this area to support this hypothesis. The detailed topology of the Africa 3 lineage was to a certain level unstable, most probably due to the low number of nucleotide sequences of African strains available in GenBank. Moreover, it was suggested previously, that Africa 3 lineage probably contains sequences of polyphyletic origin [31].

Carcasses of dead birds represent an important source of material for surveillance of USUV and other ornithophilic viruses [32,51]. As reported previously [10,14], USUV is found in multiple organs, such as liver, spleen, heart or lung. According to our results, the brain was the most suitable tissue for USUV screening compared to liver, muscle or blood, as all of the positive birds had detectable USUV RNA in the brain tissue, whereas the other tissues were negative in some of the infected individuals. Furthermore, using cell culture, we were able to isolate viable viruses even from carcasses that were for an unknown period of time (>1 day) subjected to ambient outdoor temperatures (rough estimate 15–30 °C). Mosquito C6/36 cells were highly susceptible to USUV infection, which might be associated with their deficiency in the RNA interference pathway [52]. All the USUV positive blackbirds were collected in the period of late summer (July–September) which is in accordance with the peak in large scale continual monitoring studies of birds [53] and mosquitoes [51]. No song thrushes (*T. philomelos*) were positive in our study, despite the virus which was detected in this species previously [11,32,53].

USUV was also detected in a pool of *Cx. pipiens* and in a pool of *Cx. modestus* mosquitoes. *Cx. pipiens* is considered a principal vector of USUV in Europe [1], multiple lineages were proven to be effective vectors of USUV in laboratory conditions [2,3], and there are numerous reports of detection of the virus in this vector e.g., [54,55,56]. Whereas, there is a single report of USUV found in *Cx. modestus* [30]. The authors suggest a possible role of *Cx. modestus* (together with *Cx. pipiens*) in the maintenance of USUV in a sylvatic cycle. *Cx modestus* is a human biting species and is considered a potential bridge vector of the related West Nile virus (WNV) to humans [57]. Nevertheless, it remains to be elucidated whether this species is a suitable vector for USUV, as it is for WNV [58].

In conclusion, USUV presence in the Czech Republic is not restricted to the southeastern part of the country neighboring with Austria. Two genetic lineages (Europe 1 and Europe 2) probably co-circulate in this area of endemic occurrence without causing any major outbreaks in wild life. Lineage Europe 3 was probably newly introduced to the area of Prague and resulted in a local mortality of blackbirds.

## Figures and Tables

**Table 1:** Numbers of cadavers of blackbirds (*Turdus merula*) and song thrushes (*T. philomelos*) collected in the three geographical locations in the Czech Republic, 2017–2019.

Locality	Prague	Brno			Ceske Budejovice	Total
Year	2018	2017	2018	2019	2017	
*T. merula*	8	19	13	3	12	**55**
*T. philomelos*	0	5	1	0	4	**10**
**Total**	**8**	**24**	**14**	**3**	**16**	**65**

**Table 2:** Detection of Usutu virus RNA by one-step RT-PCR in tissue samples of blackbird (*Turdus merula*) cadavers. Prevalence indicates percentage of positive samples out of tested. Not all tissue samples were available for all individuals mostly due to tissue damage. Thus, tissue efficiency is expressed as a proportion of positively tested samples of a particular tissue from all positive individuals with that tissue available.

Tissue	Prevalence (N Positive/N Tested)				Tissue Efficiency (N Positive/N Positive Individuals)
Locality	Prague ^1^	Brno	Ceske Budejovice	Total	
Brain	75% (6/8)	41% (14/34)	0% (0/12)	35% (19/54)	100% (20/20)
Liver	75% (6/8)	29% (10/34)	0% (0/11)	30% (16/53)	84% (16/19)
Muscle	75% (6/8)	31% (11/35)	0% (0/11)	30% (16/54)	84% (17/19)
Blood	75% (6/8)	17% (6/33)	0% (0/9)	24% (12/50)	66% (12/18)
Total	75% (6/8)	40% (14/35)	0% (0/12)	36% (20/55)	

^1^ Samples were acquired during local outbreak of increased blackbird mortality.

## Supplementary Materials

The following are available online at https://www.mdpi.com/2076-2607/7/11/568/s1, Table S1. Primers used for Sanger sequencing of the partial coding sequence of prM and NS4B proteins and complete coding sequences of M, E and NS5 proteins; Table S2. List of strains and nucleotide sequences of Usutu virus acquired in this study; Table S3. Detailed results of Usutu virus genomic RNA detection in the samples of blackbird (*Turdus merula*) cadavers collected 2017–2018 in the Czech Republic; Figure S1. Phylogenetic tree based on Bayesian Markov chain Monte Carlo analysis of partial NS4B coding nucleotide sequence and complete coding sequences of NS5 protein of Usutu virus; Figure S2. Phylogenetic tree based on Bayesian Markov chain Monte Carlo analysis of partial prM protein nucleotide coding sequence and complete coding sequences of M and E proteins of Usutu virus.
