# Supplementary material for: Multiple Lineages of Usutu Virus (Flaviviridae, Flavivirus) in Blackbirds (Turdus merula) and Mosquitoes (Culex pipiens, Cx. modestus) in the Czech Republic (2016–2019)
Source: Microorganisms. 2019 Nov 16;7(11):568. doi: 10.3390/microorganisms7110568 (PMC6920817; doi:10.3390/microorganisms7110568)
Supplement: Supplementary file 1 [file microorganisms-07-00568-s001.pdf]

microorganisms

Table S1. Primers used for Sanger sequencing of the partial coding sequence of prM and NS4B proteins and complete coding sequences of M, E and NS5 proteins. Some of the published primers were modified according to already obtained sequences. Modifications are indicated by lower case letter.

Region	Name	Sequence	Ann. temp.	Reference
prM/M/E protein	Usu488f	GACCATCAACGCGACTGATA	55°C	[18]
	Usu1401r	CAGAGCTGGTAGAACCATGT		
	Usu1155f	CTAGCCACTGTCTCAgATGT	50°C	[18] modified
	Usu1608r	ATGTAGTATGCCTCGGTGTT		
	Usu1326f	GACACTCATGGCAACTATTC	55°C	[52]
	Usu1892r	TCCGTACACATGCTATAGGT		
	Usu1537f	GGTTGAACACCGAGGCATAC	55°C	[18]
	Usu2505r	CTTGTCCACAGCGCAACTCT		
NS4B/NS5	Usu7314f	AGAACAGCGGCCGGAATCAT	55°C	[18]
	Usu8033r	GACAAGGTTCCAGCCATAGC		
	Usu7757f	AAGAGGCCATCACTGAAGTC	55°C	[18]
	Usu8599r	TTCCGTGGTAGGTCCAGGTC		
	Usu8499f	ACATaCCAAtCAGGAGAAGA	55°C	[52] modified
	Usu9213r	TTCAACACCTCCTCCAGAAT		
	Usu9170f	AGGACCATTGGTTAGGAAGA	57°C	[18]
	Usu9704r	GGCTTGACAACACAATCATC		
	Usu9600f	GCTGTGAGAACCTGGCTCTT	50°C	[18], Usu10292r newly designed
	Usu10592r	GCAGCACCGTCTACTCAACT		

Table S2. List of strains and nucleotide sequences of Usutu virus acquired in this study.

**Sample id. nr.**	**Year of collection**	**Area of collection**	**Source**	**prM/M/E Genbank acc. nr.**	**NS4B-NS5 Genbank acc.nr.**	**Strain identity number in BCCO collection***
019TM10	2017	Brno	*Turdus merula*	MN395372	MN419896	
025TM10	2017	Brno	*Turdus merula*	MN395373	MN419897	BCCO 50_0484 - 85
026TM10	2017	Brno	*Turdus merula*	MN395374	MN419898	BCCO 50_0486 - 87
033TM10	2017	Brno	*Turdus merula*	MN395375	MN419899	BCCO 50_0488 - 89
037TM10	2017	Brno	*Turdus merula*	MN395375	MN419900	
042TM10	2017	Brno	*Turdus merula*	MN395376	MN419901	
106TM10	2017	Brno	*Turdus merula*	MN395377	MN419902	
196TM10	2018	Brno	*Turdus merula*	MN395378	MN419903	
197TM10	2018	Brno	*Turdus merula*	MN395372	MN419904	BCCO 50_0480 - 81
200TM10	2018	Brno	*Turdus merula*	MN395379	MN419905	BCCO 50_0490 - 91
202TM10	2018	Brno	*Turdus merula*	MN395379	MN419906	BCCO 50_0492 - 93
205TM10	2018	Prague	*Turdus merula*	MN395380	MN419907	BCCO 50_0494 - 95
206TM10	2018	Prague	*Turdus merula*	MN395381	MN419908	BCCO 50_0496 - 97
207TM10	2018	Prague	*Turdus merula*	MN395380	MN419909	BCCO 50_0498 - 99
208TM10	2018	Prague	*Turdus merula*	MN395380	MN419910	BCCO 50_0500 - 01
209TM10	2018	Prague	*Turdus merula*	MN395382	MN419911	
210TM10	2018	Prague	*Turdus merula*	MN395383	MN419912	
264TM10	2017	Brno	*Turdus merula*	MN395384	MN419913	
001TM10Bre	2018	Břeclav	*Turdus merula*		MN384964	
2Cx_136	2018	South Bohemia	*Culex pipiens*	MN395370	MN395369	
3Cx16-99	2016	South Moravia	*Culex modestus*	MN395371	MN419895	

*Biology Centre Collections of Organisms: Collection of Arboviruses

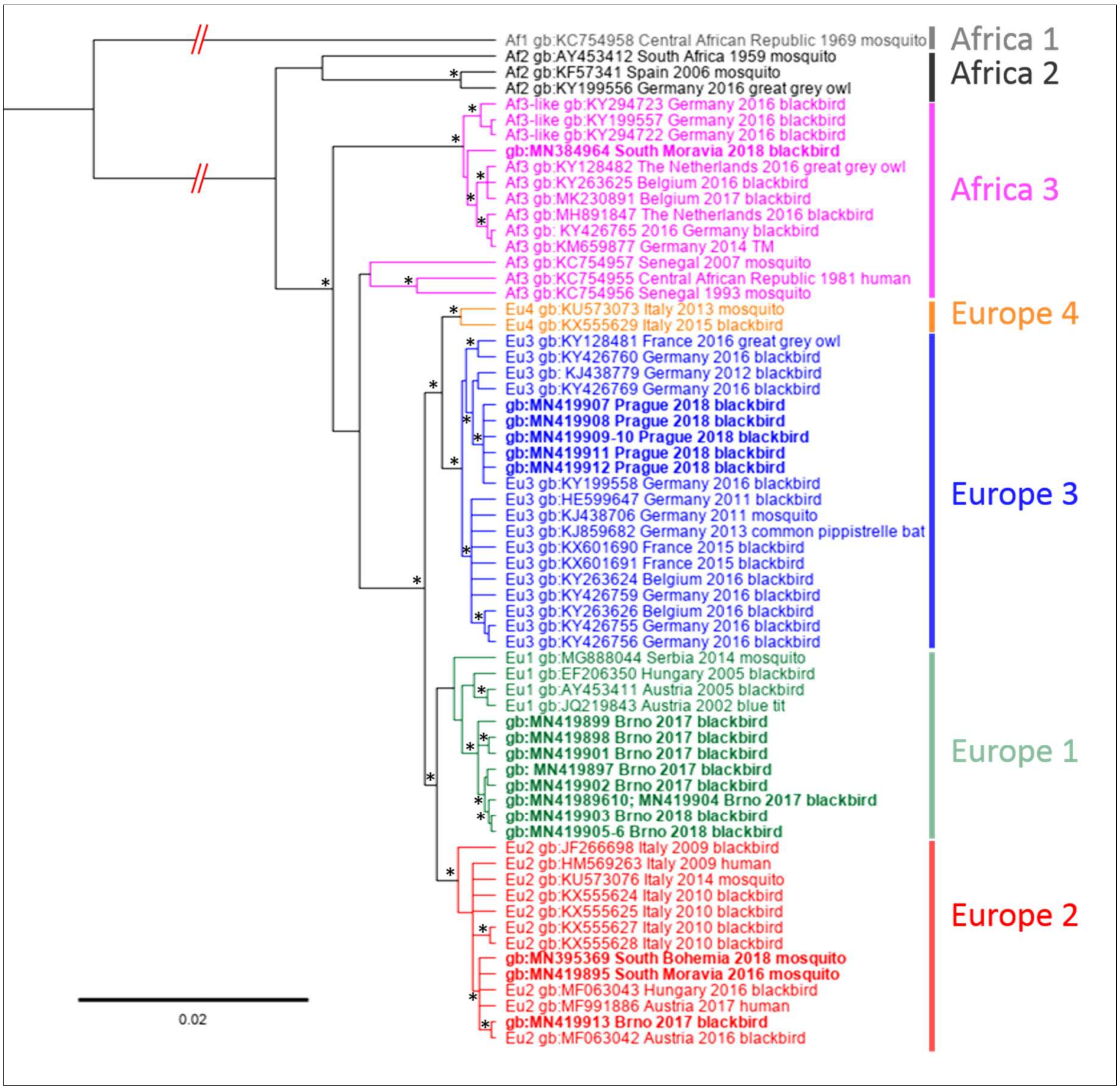

Figure S1. Phylogenetic tree based on Bayesian Markov chain Monte Carlo analysis of partial NS4B coding nucleotide sequence and complete coding sequences of NS5 protein of Usutu virus (nucleotide positions 7372-10,398 according to complete genome sequence of Vienna strain, AY453411). Sequence Africa 1, AY453412 was used as outgroup. The code of the sequences consists of GenBank accession number, place and year of origin. The sequences obtained in this study are indicated by bold, individual geographical lineages are color-coded. The tree was generated using GTR+I+G substitution model and 1,000,000 generations. Nodes with Bayesian posterior probability of >95% are indicated by asterisk. The lengths of tree branches correspond to the number of substitutions per site.

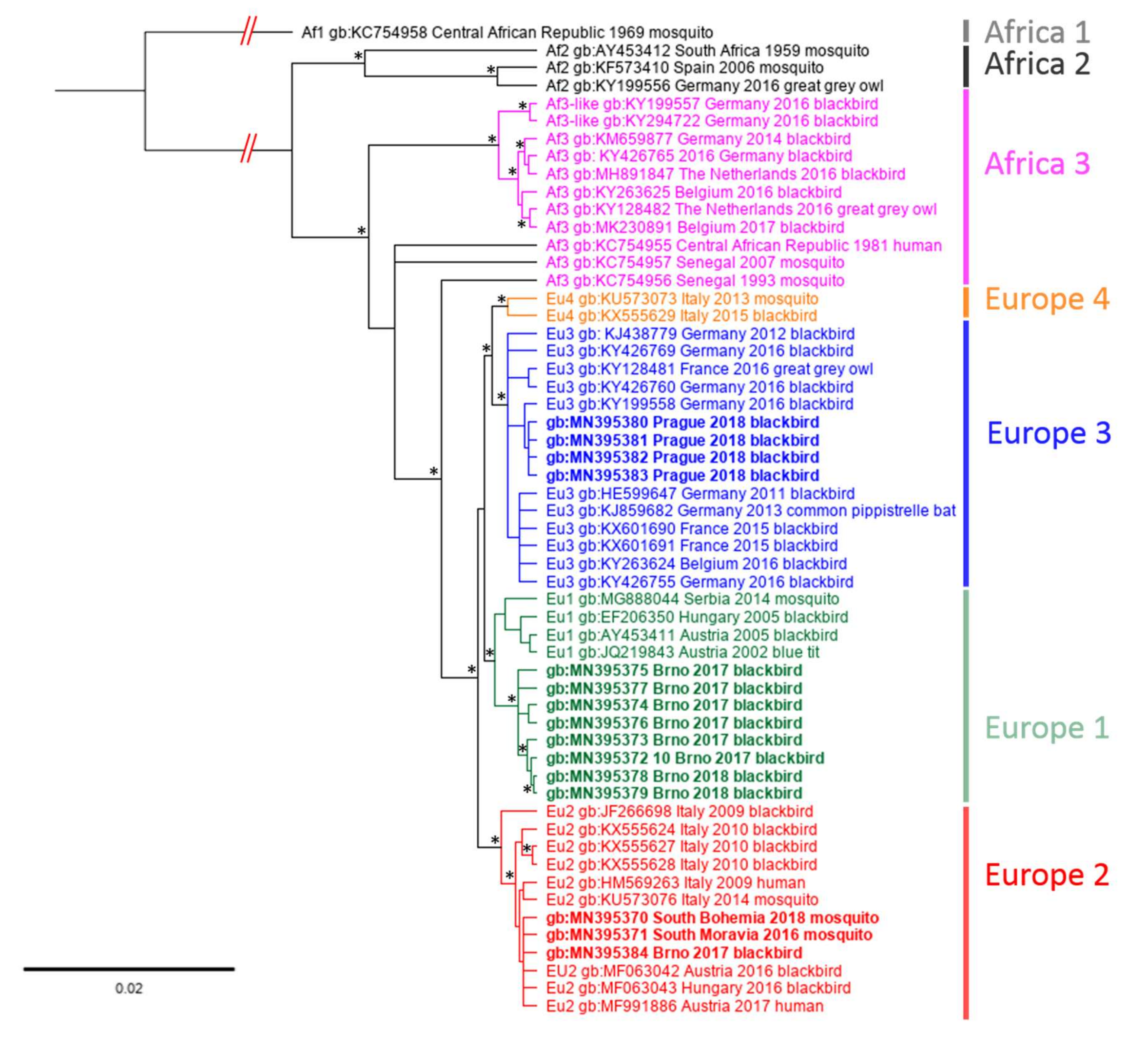

Figure S2. Phylogenetic tree based on Bayesian Markov chain Monte Carlo analysis of prM protein and complete coding sequences of M and E proteins of Usutu virus (nucleotide positions 562-2475 according to complete genome sequence of Vienna strain, AY453411). Sequence Africa 1, AY453412 was used as outgroup. The code of the sequences consists of GenBank accession number, place and year of origin. The sequences obtained in this study are indicated by bold, individual geographical lineages are color-coded. The tree was generated using GTR+I substitution model and 1,000,000 generations. Nodes with Bayesian posterior probability of >95% are indicated by asterisk. The lengths of tree branches correspond to the number of substitutions per site.
